# Supplementary figures and images for: Physiological Investigation and Transcriptome Analysis Reveals the Mechanisms of Setaria italica’s Yield Formation under Heat Stress
Source: Int J Mol Sci. 2024 Mar 9;25(6):3171. doi: 10.3390/ijms25063171 (PMC10970627; doi:10.3390/ijms25063171)

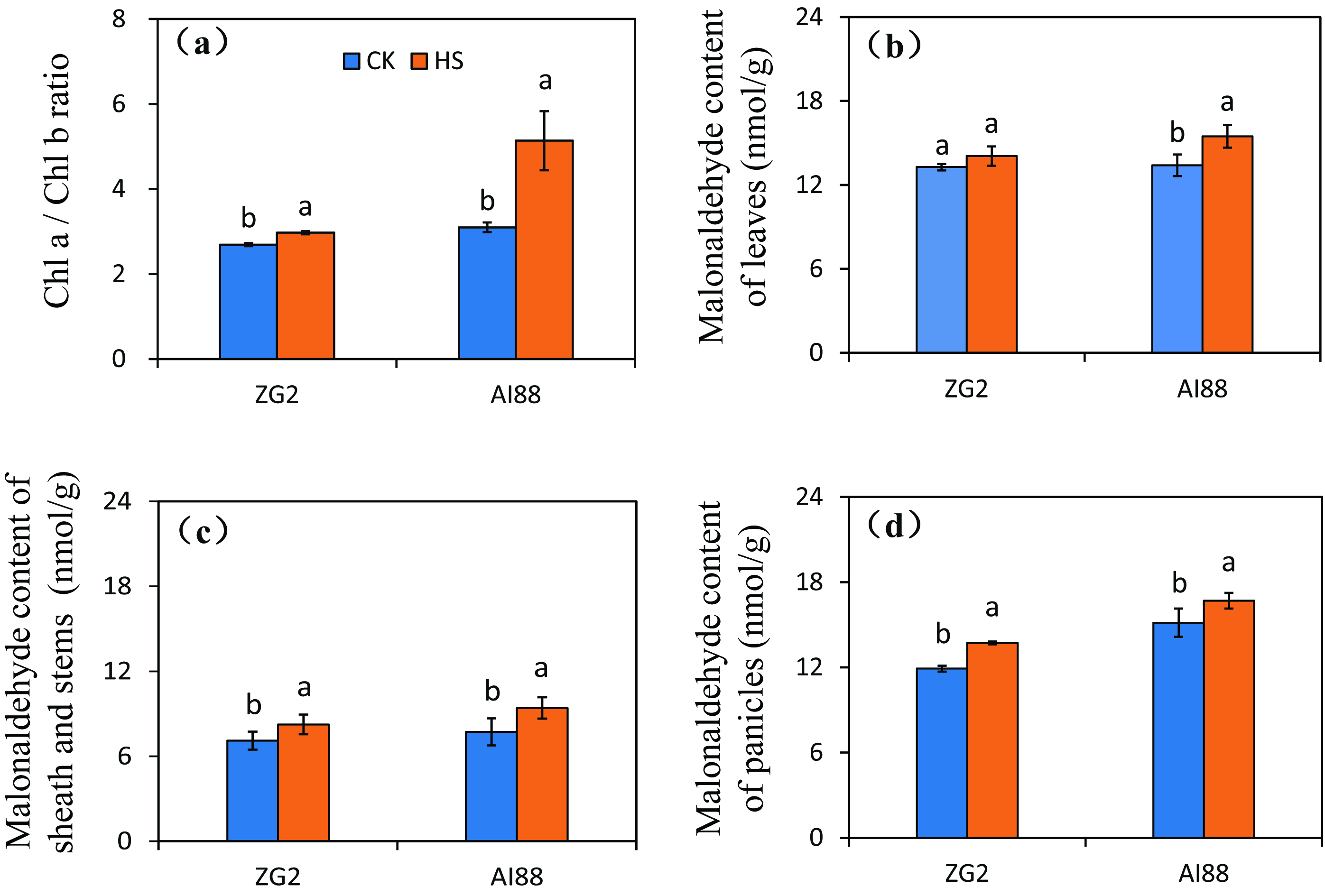

Supplement: Supplementary file 1 [file ijms-25-03171-s001.zip › Figure S1.tif]

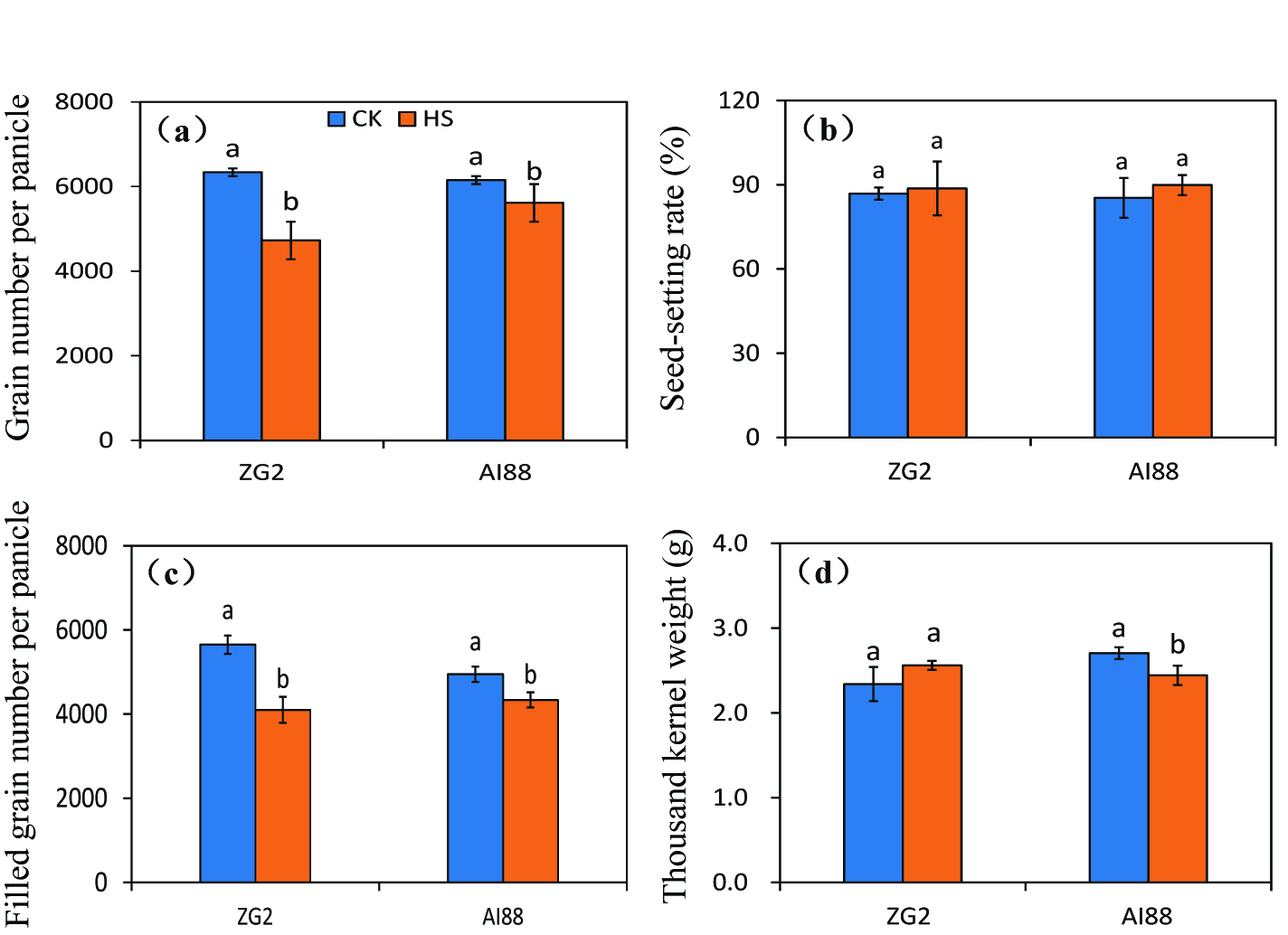

Supplement: Supplementary file 1 [file ijms-25-03171-s001.zip › Figure S2.tif]

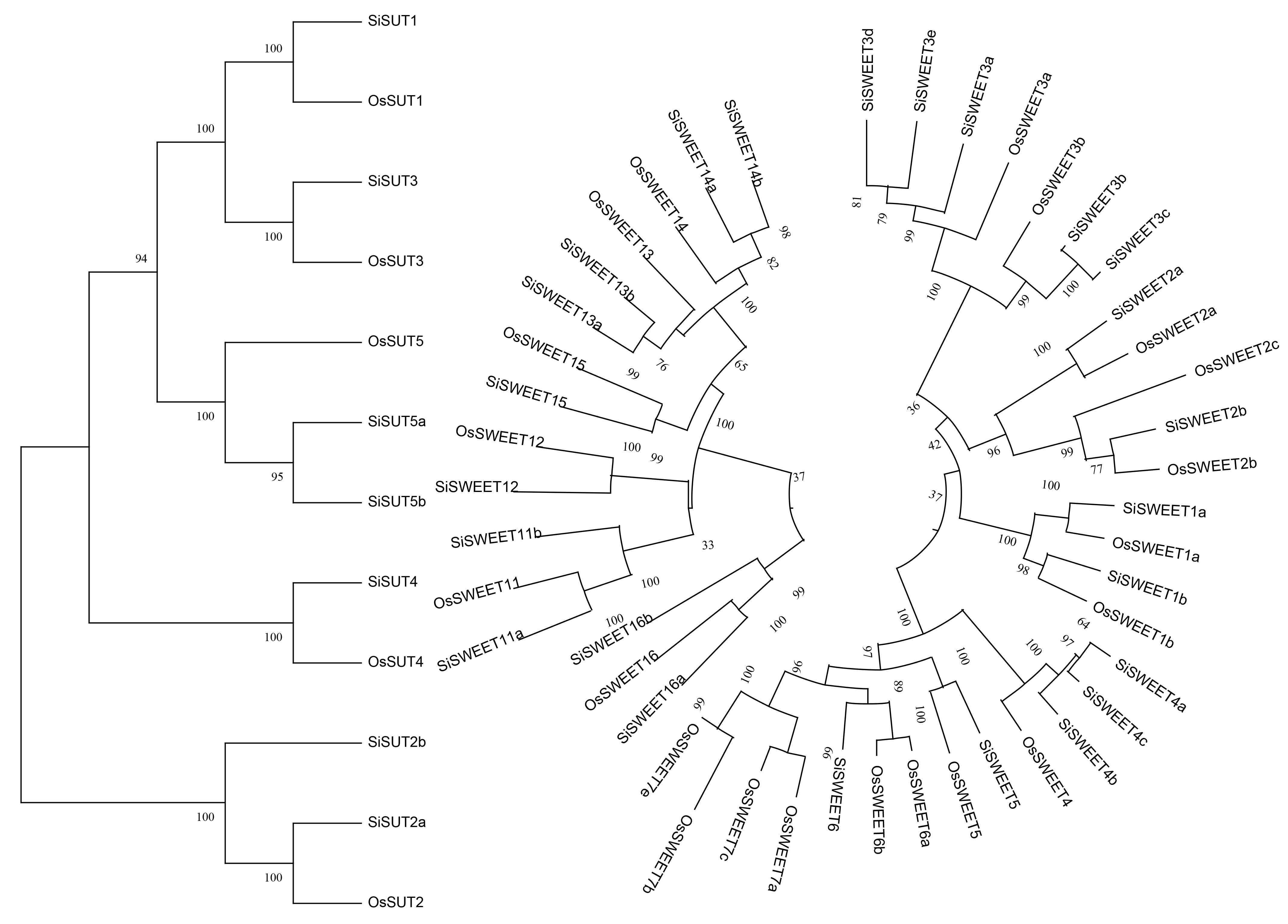

Supplement: Supplementary file 1 [file ijms-25-03171-s001.zip › Figure S3.tif]
